# Canakinumab treatment in patients with colchicine-resistant familial mediterranean fever: a multicenter observational study

**DOI:** 10.55730/1300-0144.6133

**Published:** 2025-09-30

**Authors:** Emrah KOÇ, Mete PEKDİKER, Mete KARA

**Affiliations:** 1 Division of Rheumatology, Department of Internal Medicine, Faculty of Medicine, University of Health Sciences, Adana City Training and Research Hospital, Adana, Turkiye; 2 Division of Rheumatology, Department of Internal Medicine, Faculty of Medicine, Hatay Mustafa Kemal University, Hatay, Turkiye; 3 Division of Rheumatology, Department of Internal Medicine, Faculty of Medicine, Izmir Bozyaka Training and Research Hospital, İzmir, Turkiye

**Keywords:** Amyloidosis, creatinine, familial Mediterranean fever, interleukin-1 antagonist, proteinuria

## Abstract

**Background/aim:**

Anti-interleukin-1 agents have known beneficial effects in the treatment of colchicine-resistant familial Mediterranean fever (cr-FMF); however, studies to date have tended to have small sample sizes and to be based on pooled data. The present study investigates the efficacy of Canakinumab (CAN) in a homogeneous cohort of cr-FMF cases.

**Materials and methods:**

The study included patients who underwent treatment in three tertiary rheumatology departments, whose electronic medical records were reviewed retrospectively. The inclusion criteria were presence of colchicine resistant disease activity or persistent proteinuria secondary to AA-amyloidosis, and treatment with CAN for at least 6 months. Clinical and laboratory parameters were assessed before and after CAN treatment.

**Results:**

The study included 65 patients with a mean age of 38.2 ± 13.8 years, with a mean disease duration of 23.7 ± 11.4 years and a mean colchicine dosage of 1.5 ± 0.6 mg/day. Of the total, 60% of the patients had an M694V homozygous mutation, and 41.5% were resistant to Anakinra. Furthermore, 25 had FMF-related amyloidosis, and 16 were renal transplant recipients. The mean CAN treatment duration was 31.3 ± 23.1 months, and 80% of patients achieved complete remission, while 20% achieved partial remission. Erythrocyte sedimentation rate, C-reactive protein, frequency of attacks, and patient global assessment decreased significantly after CAN (p < 0.001 for each). The mean serum creatinine level (mg/dl) decreased from 2.1 ± 0.8 to 1.4 ± 0.8 (p < 0.001), and median proteinuria (mg/day) decreased from 1475 to 675 (p < 0.001) in patients with AA-amyloidosis. Only one patient with chronic monoarthritis affecting the wrist discontinued CAN due to insufficient arthritis relief.

**Conclusion:**

Canakinumab demonstrates excellent efficacy and favorable safety as a treatment for cr-FMF. Our study is the first to indicate the efficacy of CAN in reducing serum creatinine levels.

## 1. Introduction

Familial Mediterranean fever (FMF) is the most common auto-inflammatory disease (AID), characterized by recurrent attacks of fever, serositis (pleuritis, peritonitis, pericarditis), arthritis/arthralgia, and erysipelas-like erythema. Historically, FMF is most prevalent in the Middle Eastern region, and primarily affects Turks, Armenians, Arabs, and non-Ashkenazi Jews [1]. The prevalence of FMF has been reported in the range of 1/250–500 in non-Ashkenazi Jews, and 1/1000 in Turks overall, but 1/395 in Central Anatolia [2–4]. FMF is a monogenic autosomal recessive disease caused by mutations in the MEFV gene [5, 6], which is located on the short arm of chromosome 16 (16p13.3), and encodes an inflammasome control protein known as “pyrin”. Gene mutations in pyrin lead to uncontrolled inflammation mediated by the innate immune system, with interleukin-1β (IL-1β) playing a pivotal role in the pathogenesis of FMF [7]. The pathogenesis of FMF, however, is complex, as both non-MEFV genes and environmental risk factors are also involved [8, 9].

AA-amyloidosis is a life-threatening complication of chronic inflammatory diseases, in which insoluble amyloid fibrils composed of misfolded proteins accumulate, leading to internal organ dysfunction. Rheumatic diseases such as rheumatoid arthritis, ankylosing spondylitis, and AIDs such as FMF are the most common etiological factors in patients with AA-amyloidosis [10]. AA-amyloidosis is the most serious complication and the leading cause of death in FMF, contributing to proteinuria and progressive renal failure. Colchicine is the cornerstone treatment for FMF, associated with decreased frequency and severity of attacks, as well as decreased risks of AA amyloidosis, renal failure, and premature death [11]. The incidence of AA-amyloidosis in patients with FMF was 60% prior to the advent of colchicine, but decreased to 8.6% after colchicine treatment [12, 13]. European Alliance of Associations for Rheumatology (EULAR) recommends starting colchicine treatment as soon as possible when a patient is diagnosed with FMF [14].

It should be noted that 5%–10% of patients with FMF do not respond to colchicine treatment, despite regular high dosages [15]. Furthermore, colchicine is contraindicated in 10%–20% of patients due to side effects [16]. When the role of IL-1 in the pathogenesis of FMF began to be understood, biologic agents targeting IL-1 started to be used in colchicine resistant-FMF (cr-FMF) patients. Canakinumab (CAN) is a monoclonal antibody against IL-1β, while anakinra (ANA) is an IL-1 receptor antagonist, and both have been approved for use in the treatment of patients with cr-FMF [17].

A Science Citation Index Expanded-based bibliometric analysis reveals Türkiye to be the country that has published the most studies of FMF [17]. A meta-analysis from Türkiye reported anti-IL-1 to be effective and safe for the treatment of pediatric and adult patients with cr-FMF. However, the published studies from Türkiye focusing on anti-IL1 treatment in patients with cr-FMF are limited by their small sample sizes, their single-centered design, and their use of pooled (both ANA and CAN) data [18]. The present multicenter study aims to overcome these limitations by investigating the efficacy and safety of canakinumab in a large, homogeneous cohort of patients with cr-FMF, representing one of the largest case series focused solely on this biologic agent.

## 2. Materials and methods

### 2.1. Patient selection

Included in this retrospective observational study were adult patients treated at three tertiary rheumatology departments (namely, Hatay Mustafa Kemal University Faculty of Medicine, Izmir Bozyaka Training and Research Hospital, and Adana City Education and Research Hospital) in Türkiye between January 2020 and January 2025. The inclusion criteria were as follows: fulfilling the Tel-Hashomer classification criteria [19]; presence of colchicine-resistant disease activity according to the EULAR definition [14] or persistent proteinuria secondary to FMF-related AA-amyloidosis; receiving a regularly administered maximum tolerable dose of colchicine for 6 months, defined as a minimal dosage of 1.5 mg/day (in the absence of chronic kidney disease) or 1 mg/day (in the presence of chronic kidney disease) for at least 6 months; and receiving regular 150 mg doses of CAN every 4 weeks for at least 6 months.

Excluded from the study were patients with irregular follow-ups; gaps in laboratory or treatment data; CAN treatments of less than 6 months; and use of any immunosuppressive drugs (such as azathioprine, mycophenolate mofetil, calcineurin inhibitors, prednisolone ≥ 7.5 mg/day) concomitantly, except for those required after renal transplantation while undergoing CAN treatment. The biologic agent (ANA or CAN) provided to the patients with cr-FMF depended on off-label approval from the Ministry of Health. If patients with cr-FMF were found to be resistant to ANA 100mg/day for a period of 3 months, they were switched to CAN.

The retrospective nature of this study opens it up to the risk of potential selection and reporting biases. To minimize these biases, the electronic records of all centers were systematically screened according to predefined criteria and patients were enrolled consecutively. Patients with missing data were excluded from the study.

A flow diagram of patient selection is presented below:

**Figure:**
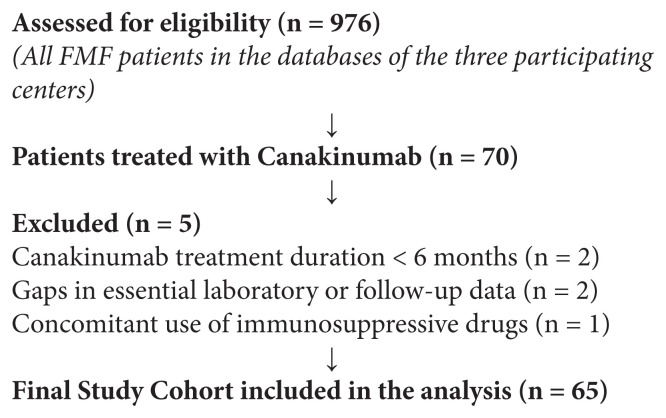


### 2.2. Assessments

The electronic medical files were reviewed retrospectively. The age, sex, disease duration, age at disease onset, lag time to diagnosis, secondary rheumatic disease other than FMF, frequency and clinical features of FMF attacks, and laboratory and treatment data of the patients were noted at baseline. MEFV gene mutations were analyzed from venous blood samples by real time polymerase chain reaction; exons 2, 3, 5, and 10 were analyzed. The patients’ complete blood counts, biochemical profiles, and acute phase reactants, including erythrocyte sedimentation rate (ESR) and C-reactive protein (CRP), were evaluated from peripheral serum samples at baseline and then every 3 months during the follow-up attack-free period. Complete urinalysis, and 24-h urine protein excretion in those with AA-amyloidosis were evaluated at baseline during attack-free periods, and every 3 months during follow-up. Chronic kidney disease (CKD) was defined as having a glomerular filtration rate < 60mL/min/1.73 m^2^, and persisting for more than 3 months. AA-amyloidosis was diagnosed by renal biopsy showing Congo red, and immunohistochemically confirmed amyloid-A staining. FMF disease activity was evaluated by patient global assessment (PGA) using a visual analog scale (VAS: 0 cm = no symptoms, 10 cm = most severe symptoms). Baseline, and final acute phase reactants, frequency of attacks in the last six months, and VAS scores were compared.

The number of prescriptions for colchicine received from physicians during the previous year and the number of these prescriptions that were subsequently filled were ascertained. As both the prescribing and the filling of prescriptions were fully computerized, the data was readily available.

The effectiveness of CAN was defined as either complete remission (no attack) or partial remission (1–3 attacks over a 6-month period). All patients resumed the maximal tolerated dose of colchicine concomitantly during biologic therapy. Canakinumab was started at a standard dose regimen of 150 mg every 4 weeks, and after the first 12 months, the CAN dosing interval was extended to 150 mg every 8 weeks in the event of partial or complete remission with the standard dosing regimen. Patients with renal transplantation continued their regular immunosuppressive therapies, including corticosteroids, mycophenolate mofetil, and calcineurin inhibitors. Patients with purified protein derivative test results of 0 mm or ≥ 5 mm, or a positive QuantiFERON test result received isoniazid 300 mg/day for 9 months for tuberculosis prophylaxis. Death, infectious events requiring intensive care unit admission or hospitalization, active tuberculosis, hepatitis B virus reactivation, disseminated zoster infection, and immune-mediated side effects requiring discontinuation of CAN were defined as serious adverse events (SAEs). Injection site reactions were noted if they occurred. The final assessment was made in January 2024. Treatment response was assessed based on the following pre-defined criteria: complete remission, defined as the absence of any FMF attacks over a six-month period; or partial remission, defined as experiencing between 1 and 3 attacks over a 6-month period.

## 3. Statistical analysis

The data obtained in the study were analyzed using the IBM SPSS Statistics Version 20.0 (IBM Corp., 2011). The suitability of variables for normal distribution was determined by a Kolmogorov–Smirnov test. Continuous variables with normal distribution were expressed as mean ± standard deviation, while those without normal distribution were expressed as median (min–max). Categorical variables were expressed as numbers and percentages. Student’s t-test was used for continuous variables with a normal distribution, and the Mann-Whitney U-test for those without a normal distribution. The Chi-square test was used to evaluate categorical variables. Comparisons between dependent groups were made with a Friedman test or ANOVA. p values less than 0.05 were considered statistically significant. No correction for multiple comparisons was applied (e.g., Bonferroni) as the study was considered exploratory in nature, and the intention was to avoid increasing the type II error rate given the sample size. All participants gave written informed consent for the use of their data in the study. The local ethical committee of the University approved the study (date: 12.25.2023, number: 21).

## 4. Results

### 4.1. Characteristics of the study group

The records of 976 patients with FMF were reviewed retrospectively. Of the 70 patients who were treated with CAN, 65 were enrolled, while five were excluded due to the short duration of CAN treatment (n = 2), lack of data (n = 2), and concomitant use of immunosuppressive drugs (n = 1). Of the total, 58.5% (n = 38) of the patients were male, and the mean age was 38.2 ± 13.8 years. The median lag time to diagnosis was 12 months, the mean disease duration was 23.7 ± 11.4 years, the mean number of attacks in the 6 months before starting CAN treatment was 6.8, and 43% of patients had a family history of FMF. The rates of AA-amyloidosis, renal transplantation, CKD, and end-stage renal disease (ESRD) were 38.5%, 24.6%, 6.1%, and 3%, respectively. Among the patients, three had chronic hip monoarthritis, one had chronic wrist monoarthritis, and one had IgA vasculitis as secondary rheumatic diseases. The mean colchicine dosage was 1.5 ± 0.6 mg/day; the colchicine dosage was 0.5 mg/day in patients with ESRD and 1 mg/day in those with CKD. Furthermore, 50.7% of the patients (n = 33) had used ANA before CAN, 27 of whom had discontinued ANA due to a lack of benefit, while six quit due to injection site reactions. The most common MEFV gene mutation was homozygous M694V, present in 60% (n = 39) of the patients. Of the total, 78.5% (n = 51) had at least one M694V mutation and two patients had a negative test result for MEFV gene mutations. All patients had high CRP values before starting treatment with CAN.

Table 1 and Table 2 show the demographic, laboratory, clinical, and treatment characteristics of the study population.

### 4.2. Efficacy of Canakinumab

The patients used CAN for a mean duration of 31.3 ± 23.1 months, achieving overall excellent efficacy, with 80% (n = 52) and 20% (n = 13) of the patients achieving complete and partial remission, respectively. CAN was also found to be effective in patients with amyloidosis, with 14 of 25 patients achieving complete remission and the remaining 11 achieving partial remission. The mean ESR and CRP significantly decreased after CAN (p < 0.001, p < 0.001), with CRP normalizing in 55 patients. The median PGA (cm) decreased significantly from 8 to 0 (p < 0.001), and the median number of attacks every 6 months decreased significantly from 7 to 0 after CAN (p < 0.001).

The mean serum creatinine decreased from 2.1 ± 0.8 mg/dL to 1.4 ± 0.8 mg/dL (p < 0.001), and the median 24-h urine protein excretion decreased from 1475 mg to 675 mg (p < 0.001) in patients with AA-amyloidosis without ESRD (n= 23). Furthermore, three patients with chronic hip monoarthritis had no locomotor symptoms under CAN therapy, while the one patient (20-year-old male) with chronic monoarthritis affecting the wrist discontinued CAN and achieved remission with Etanercept.

Approximately half of the sample (n = 33) had used ANA before CAN. Of these, 27 discontinued ANA treatment due to insufficient response and six due to injection site reactions, and switched to CAN. Of these patients, 27 achieved complete remission, and six experienced partial remission. Table 3 shows the efficacy of CAN.

### 4.3. Safety of Canakinumab

Within the sample, one patient (65-year-old female) developed pneumonia and one (43-year-old male) had an intra-abdominal abscess, and both required hospitalization and intravenous antibiotics. Both patients fully recovered from their infections and continued their CAN treatment without any further issues. In addition, four patients developed mild injection site reactions to CAN, although none discontinued the treatment. No deaths were noted during the follow-up period

## 5. Discussion

The most significant and novel finding of this study is that canakinumab significantly reduced serum creatinine and proteinuria levels in patients with FMF-related amyloidosis, independent of their baseline serum creatinine levels. This contrasts with some previous lower level evidence studies that reported no significant effect on creatinine [23, 26]. To the best of our knowledge, this is the first large case series in the literature to clearly demonstrate this beneficial renal effect of canakinumab. This observational multicenter study analyzing the efficacy and safety of CAN in patients with cr-FMF represents the largest cohort to date, encompassing patients with cr-FMF, ANA-resistant FMF, FMF-related AA-amyloidosis, and FMF-related renal transplantation treated with CAN. The study found CAN to be effective in decreasing ESR, CRP, PGA, number of attacks, and 24-h urine protein excretion, and for the first time in the literature, that serum creatinine significantly decreased after CAN, regardless of the baseline serum creatinine level in patients with FMF-related amyloidosis.

The interpretation of this striking reduction in serum creatinine and proteinuria warrants caution. While our results are promising, the observational nature of our study cannot fully exclude the possibility of “regression to the mean” – a statistical phenomenon in which extreme initial measurements naturally tend to move closer to the average in subsequent readings – or the influence of other concomitant medications and supportive care. Nevertheless, the consistent and significant improvement across a substantial number of patients, coupled with the reduction in acute-phase reactants, strongly suggests a genuine treatment effect. The most plausible mechanism for this renal benefit is the potent suppression of IL-1β by canakinumab, which may halt the continued inflammatory drive and subsequent amyloid deposition responsible for renal dysfunction (preventing the so-called “amyloid storm” [24]), thereby potentially allowing for some degree of functional recovery. This anti-inflammatory effect may also reduce glomerular and tubular damage, leading to improved filtration and reduced protein leakage.

In a study by Karabulut Y. et al. involving the second highest number of cr-FMF cases in the medical literature (n = 57), 70% of the cohort were homozygous for the M694V mutation, and the authors reported CAN to be effective in decreasing ESR, CRP, frequency of attacks, duration of attacks, and disease severity. In their previous study, the dosing interval of CAN was extended in 22 of 57 patients, after which, nine had no attacks and 10 had a drug-free period without attacks for 6 months [20]. In our study, 50 of the 65 patients received CAN 150 mg for 8 weeks after first 12 months, and none experienced a worsening in disease activity. In a study by Atas N. et al. involving 101 participants, 56% were homozygous for the M694V mutation, and one-third had amyloidosis. The authors reported a significant decrease in the number of attacks, VAS scores, levels of CRP, and ESR after anti-IL-1 therapy, while CAN showed no effect on decreasing proteinuria [21].

In a study by Sahin A. et al. conducted with 65 patients with cr-FMF, both ANA (n = 42) and CAN (n = 23) were found to be beneficial in reducing the number and duration of attacks, as well as CRP, ESR, and VAS scores, while no effect on proteinuria was noted [22]. The Turkish FMF Multi-centered Investigations Platform in Rheumatology carried out a study of 172 cr-FMF patients treated in 21 centers, 31% of whom had amyloidosis. The researchers reported anti-IL-1 agents to be effective in reducing the frequency and duration of attacks, levels of CRP, ESR, and 24-h urine protein excretion, but to have no effect on serum creatinine. The study included 21 patients using CAN, but a subgroup analysis of patients treated specifically with anti-IL-1 agents was lacking [23]. The 80% complete remission rate achieved with canakinumab in our cohort would appear to be higher than that reported in previous larger studies, including some analyzing anakinra and canakinumab together or including less homogeneous groups. For instance, in a nationwide study by Akar et al., a response rate (complete and partial response combined) of 88% was reported with anti-IL-1 agents, while the study by Sahin et al. recorded a complete response rate of 61.5% in their canakinumab subgroup [22, 23]. Our high remission rate may be attributed to the exclusive use of canakinumab and the relatively long treatment duration in our study.

In a study by Ugurlu S. et al. investigating the efficacy of anti-IL-1 agents in 44 patients with FMF-related amyloidosis, anakinra was discontinued in nine patients and CAN in five patients in the study due to a worsening of renal function. The authors found that anti-IL-1 agents could be considered safe and effective (regarding kidney function, proteinuria, and acute phase reactants) for the treatment of patients with FMF-related amyloidosis, especially those with serum creatinine levels < 1.5 mg/dL [25]. The authors also reported an increase in serum creatinine levels in cases with baseline serum creatinine levels of > 1.5 mg/dL. In our study population, 18 patients with amyloidosis (15 of them with serum creatinine levels ≥ 1.5 mg/dL) were given CAN for the treatment of persistent proteinuria, and all experienced decreases in serum creatinine levels and 24-h urine protein excretion, regardless of their baseline serum creatinine level. Varan O. et al. evaluated the efficacy of anti-IL-1 agents in patients with FMF-related amyloidosis (n = 16) in a study in which six of the 16 patients were treated with CAN and 10 with ANA. The authors found that ESR, CRP, VAS, and 24-h urine protein excretion to be significantly decreased after anti-IL-1 therapy, while serum creatinine was not [26].

The efficacy and safety of canakinumab were apparent in the 16 kidney transplant recipients (KTRs) in our study, concurring with previous case reports focused on this subject and a systematic review of CAN in KTRs [27].

In the present study, four of the 65 patients had chronic monoarthritis, affecting the hip (n = 3) and wrist (n = 1), and CAN was shown to be effective for the treatment of hip arthritis, while the patient with chronic wrist monoarthritis was switched to Etanercept. Kehribar DY. et al. reported the efficacy of anti-IL-1 agents in patients with FMF-related chronic arthritis in 18 patients, among whom 16 achieved remission after starting treatment with ANA (n = 10) and CAN (n = 6) [28]. Almost 5% of FMF patients develop chronic arthritis, which is strongly associated with the M694V mutation and usually does not respond to colchicine treatment [29, 30]. While there is a lack of consensus on the optimum treatment, EULAR recommends disease-modifying antirheumatic drugs (DMARDs), intra-articular steroid injections or biologics [14].

Despite the remarkable results of the present study, there are several limitations that should be taken into account. First, its retrospective design inherently carries risks of selection and reporting bias. Second, serum amyloid-A (SAA) levels – a key biomarker for amyloidosis and FMF activity – were not investigated, even though serum CRP levels are known to be well-correlated with SAA in patients with FMF. Third, the absence of serum amyloid P component (SAP) scintigraphy, considered the optimum approach to the evaluation of the extent of systemic amyloidosis [30], can be considered a further limitation. Fourth, the variability in observation and treatment durations (6–110 months) may lead to bias in the assessment of long-term treatment outcomes. Finally, the lack of a randomized control arm prevents any definitive conclusions being drawn regarding comparative efficacy. Among the strengths of the study are its analysis of the largest case series to date focusing on the efficacy of CAN in a homogeneous cohort of patients with cr-FMF, including those with ANA-resistant FMF, FMF-related amyloidosis, and KTRs secondary to amyloidosis.

Our specific focus on CAN has contributed to the collection of homogeneous and robust data on its use in cr-FMF. Our findings regarding the improvement of renal parameters are particularly noteworthy, and suggest that canakinumab may be considered a viable treatment option, especially for patients with impaired kidney function secondary to amyloidosis, potentially altering the disease course and improving outcomes. While we have shown CAN to be an effective and safe treatment option, long-term prospective studies are required to better define the efficacy and safety of biologic agents in patients with cr-FMF, amyloidosis, and KTR.

## Figures and Tables

**Table 1:** Demographic, clinical, and treatment characteristics.

Total patient, (n)	65
Male sex, n, (%)	38 (58.5)
Female sex, n, (%)	27 (41.5)
Age, mean ± SD, years (min–max)	38.2 ± 13.8 (18–72)
Age at disease onset, mean ± SD, years (min–max)	13.9 ± 9.1 (2–60)
Lag time to diagnosis, median, months (min–max)	12 (2–132)
Disease duration, mean ± SD, years (min–max)	23.7 ± 11.4 (1–54)
Fever, n, (%)	65 (100)
Peritonitis, n, (%)	57 (87.7)
Pleuritis, n, (%)	20 (30.7)
Pericarditis, n, (%)	4 (6.1)
Orchitis, n, (%)	2 (3)
Arthralgia or acute arthritis, n, (%)	31 (47.7)
Erysipelas like erythema, n, (%)	14 (21.5)
AA amyloidosis, n, (%)	25 (38.5)
Renal transplantation, n, (%)	16 (24.6)
Chronic kidney disease, n, (%)	4 (6.1)
End stage renal disease, n, (%)	2 (3)
Colchicine dose, mean±SD, mg/day (min–max)	1.5 ± 0.6 (0.5–3)
Isoniazid preventive therapy, n, (%)	6 (9.2)
Prior biologic (Anakinra) use, n, (%)	33 (50.7)
Duration of Canakinumab, mean±SD, months (min–max)	31.3 ± 23.1 (6–110)
Response to Canakinumab, n, (%)	
-Complete remission	52 (80)
-Partial remission	13 (20)
Serious adverse event, n, (%)	4 (6.2)
Canakinumab discontinuation, n, (%)	1 (1.5)

Abbreviations: SD, standard deviation; FMF, familial Mediterranean fever.

**Table 2:** MEFV gene analysis of study group.

MEFV gene mutations	Patient count (%)
M694V/M694V (homozygous)	39 (60)
M694V/- (heterozygous)	3 (4.6)
M680I/M680I	1 (1.5)
E148Q/E148Q	1 (1.5)
Compound heterozygous	
- M694V/M680I	3 (4.6)
- M694V/V726A	3 (4.6)
- M694V/E148Q	2 (3.1)
- M694V/R202Q	3 (4.6)
- M680I/V726I	2 (3.1)
- E148Q/R202Q	2 (3.1)
- R202Q/G138G/A165A	1 (1.5)
- M694V/E148Q/R202Q	3 (4.6)
Negative	2 (3.1)

**Table 3:** Comparison of laboratory and disease activity parameters.

Variable	Baseline (during attacks free interval)	Final	P value
ESR, mean ± SD (min–max), mm/h	63.3 ± 17.7 (13–95)	14.9 ± 10.4 (2–44)	<0.001
CRP, mean ± SD (min–max), mg/L	95.2 ± 65.6 (15–309)	4.1 ± 3.1 (0.5–18)	<0.001
PGA, median (min–max), cm	8 (6–10)	0 (0–6)	<0.001
Number of FMF attack in last six months, median ± SD (min–max)	7 (5–10)	0 (0–2)	<0.001
Serum creatinine, mean ± SD (min–max), mg/dL (n = 23)	2.1 ± 0.8 (1.2–4.5)	1.4 ± 0.8 (0.6–4.0)	<0.001
24 h urine protein excretion, median (min–max), mg (n = 23)	1475 (450–21,000)	675 (150–6500)	<0.001

Abbreviations: ESR, erythrocyte sedimentation rate; CRP, C-reactive protein; PGA, patient global assessment; FMF, familial Mediterranean fever; SD, standard deviation.

## Data Availability

The data underlying this article can be obtained from the corresponding author upon reasonable request.
